# Supplementary material for: Discourses of change: The shift from infibulation to sunna circumcision among Somali and Sudanese migrants in Norway
Source: PLoS One. 2022 Jun 17;17(6):e0268322. doi: 10.1371/journal.pone.0268322 (PMC9205475; doi:10.1371/journal.pone.0268322)
Supplement: S3 File — (DOCX) [file pone.0268322.s003.docx]

**FOR ADULTS (16-100 talking about themselves)**

1. Background information: nationality, childhood experience and life, parents education, work and ethnicity, level and type of education, whether grew up in rural or urban area, eventual moving between different locations during childhood and youth age.
   1. What are the factors that mostly affect maintenance or change with regards to these practices: religion, culture, traditional or modern values?
2. (Introduction to the topic): What was your first exposure to or experience with the topic of female circumcision? Heard something, seen something, experienced something, discussions
   1. Have you been involved in a situation where there has been pressure to cut/not cut between relatives across borders (go back to drawing)
   2. What was your first experience of resistance/critical discourses on circumcision?
3. Are there significant differences between the way people think about and act with regards to female circumcision, social control and forced marriage in your (ethnic) circles in Norway, in your country of origin, or in other exile countries (e.g. Great Britain, the Netherlands, other Scandinavian countries and the middle east?)
4. Are these topics (female circumcision, social control and forced marriage) discussed across countries (your relatives, and friends in other countries)? Is change happing simultaneously or in different speed?
5. List all countries where you have close relations (call regularly, could visit, could help)
6. For married women: how they met their husbands (family approval, contact)
7. How is it to mother / raise a daughter without circumcision? A girl who is different from you

Optional:

What do you remember from your own circumcision? Who decided? Who performed the procedure? Any complications? Alone or with others. How was it done (“glue(mal mal) stiches, tied legs, healing period, movement restrictions).

**FOR YOUNG GIRLS**

- 1. Are you (and friends/relatives) experiencing stigma (harassment, bullying, or pressure with regards to female circumcision in Norway? (from Norwegians or others who don’t have these traditions, or from your own community that has. Harassed because having circumcision, or because not having it – in different contexts (Norway, visiting family abroad, visit or from relations in country of origin)
  2. Have you been visiting your country of origin or relatives in another country of migration? Where, when and why?
  3. Have you been bullied or pressurized for not being circumcised, or for being cut?
  4. Have you experienced pressure or bullying regarding surgical treatment of circumcision (pressure to not defibulate, to reinfibulate or against clitoral reconstruction?
  5. Concerning who to marry? Limits on our choices? Stigma or pressure?
  6. The fact that you are/have not undergone (Pick what suites) affect how you think about who you can marry or not? (e.g. if you have FGC, are you concerned that a partner that has not grown up with this tradition would have challenges accepting it? Or if you don’t have FGC (or if not pharaonic) would you be worried that a partner with background from a country where this is the norm would not accept your lack of FGC?)
  7. Are there any ways in which you feel family or friend try to control your behavior? For example, do you have to hide eventual boyfriend, smoking or drinking habits, dress in another way than you would like etc.?
     1. If so, do you feel this control from family or friends in Norway, or other parts of the world, e.g. home country?
  8. Do you discuss FGC, forced marriage or honor and control with family and friends who share the same ethnic background as you, but live in other countries than Norway? If so, with who? Do you feel the attitudes and concerns are the same or different in different countries? Explain?
  9. Do you feel that FGC is a part of your identity? As a somalin/Sudanese or other? Is it a part of your culture, or religion, or sense of belonging? (if you don’t have FGC, do you feel that it sets you apart from others in your ethnic group? Or if you have, that it is part of belonging?)

**FOR mother – questions regarding their daughters**

- 1. If your own daughters have FGC, do you sense that this is a private concern that should be hidden, or something that you have to prove? Explain, what situations?
  2. If you have a daughter with FGC, who decided on this? Was it with or against your will? The other parents will? The daughters will?
  3. If your daughter have pharaonic FGC, would you support her if she wants to open it/have a defibulation? At what age? For what reason would it be OK, not OK? Why? Can they have an opening surgery whiteout plans for marriage? Would you seek advice if she wanted or needed this? If so, with who? Would you discuss it with family and relatives in other countries? Would you tell them? Why? Have you experienced any such situation, or heard about it from others?
  4. If your daughters don’t have FGC, do you experience any pressure from relatives in other countries to do so? If so, what type, and what reasons, and who pressure? Would uncut girls be exposed to ridicule or harassment if they visit your country of origin? Have you experienced or heard about? Is there anything special you would have to do to protect your daughters from FGC? Or from being harassed for having/not having it? (Could you let your daughters live for months, or years, with your parents? Siblings? Others in home country without fear of FGC?
  5. What are your thoughts concerning your children’s choice of marital partner? Are there limits to their chose? What would be the best imaginable marriage partner in your view? Why? What would be the worst? Why? (ethnicity, religion, culture??) what could be done to influence children’s choice of marriage partner?
  6. Do you discuss marriage plans/partners of your children or relatives and friends children with family and friends in other countries? Are you discussing other issues, including FGC, marriage or honor? Dress code? Religion? Education? Work? (abortion? Children?)
  7. Do you consider FGC as an integral part of your culture? Tradition? Religion? Identity? Belonging?
