# Supplementary figures and images for: Discourses of change: The shift from infibulation to sunna circumcision among Somali and Sudanese migrants in Norway
Source: PLoS One. 2022 Jun 17;17(6):e0268322. doi: 10.1371/journal.pone.0268322 (PMC9205475; doi:10.1371/journal.pone.0268322)

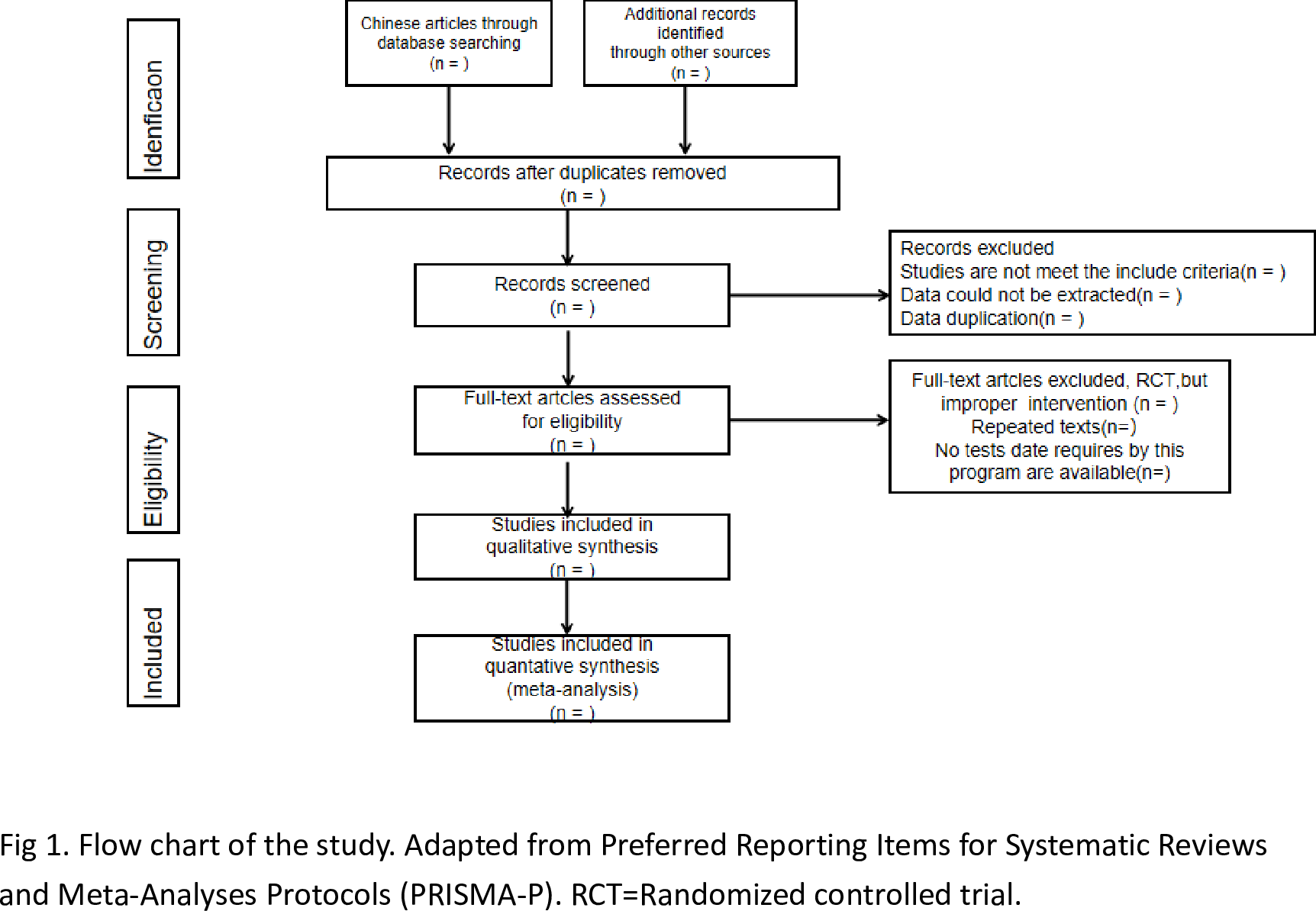

Supplement: S1 Fig — Adapted from Preferred Reporting Items for Systematic Reviews and Meta-Analyses Protocols (PRISMA-P). RCT = Randomized controlled trial. (TIF) [file pone.0268322.s004.tif]
