# Supplementary material for: Discourses of change: The shift from infibulation to sunna circumcision among Somali and Sudanese migrants in Norway
Source: PLoS One. 2022 Jun 17;17(6):e0268322. doi: 10.1371/journal.pone.0268322 (PMC9205475; doi:10.1371/journal.pone.0268322)
Supplement: Attachment — Submitted filename: Plos One reviewer comments.docx [file pone.0268322.s005.docx]

Plos One reviewer comments –

Very important and interesting paper with informative content and interesting points brought to light. However, the paper would benefit from undergoing English proof reading, sometimes the meaning gets lost due to incorrect (I assume) use of words. Also, you sometimes write ‘we’ and sometimes ‘me’.

General comments:

Introduction:

You have two aims, it would be clearer if you place these together, perhaps turn them into a broader aim and two objectives.

The result section is clear and informative.

Discussion:

The discussion is a bit messy and could benefit from clarification, could do with subheadings. I suggest you try to separate the points a bit, perhaps discuss sexuality by itself, and then tradition/religion, and then ‘modern/backwards’. I think this will provide some clarification. Also look over how you use ‘real’ and ‘ideal’ and ‘discourse’ and ‘reality’, and how these connect to each other.

Interesting discussion on religion here.

Conclusion:

I like your conclusion as your points, but not sure if the point about sexual control to move from outer (infibulation) to inner (sunna) works – except for in the discourse (if the ‘real’ sunna is closure anyway). Perhaps one point that could be highlighted more is that without the changes in meaning of female sexuality (ie that it still needs to be controlled), a change to lesser forms of FGC (or abandonment) is unlikely to take place, at least in more than the discourse. This does come across, but could be clearer.

Specific comments:

Page 3, first half: Please provide a reference to the sentence: *Nevertheless, existing evidence suggest that the change of type is both less widespread and substantial than commonly claimed.*

Look over this sentence, *Furthermore, the change of anatomical extent of FGC is associated with changes in the meaning making, in which religion seems to play a major role, while concerns over female sexuality, a major component of infibulation, still is a major cultural and religious value*. Can it be divided and clarified?

Page 4:

*Thus, the contrast between infibulation and sunna circumcision can, in some cases, be limited to a slight difference in the extent of vaginal closure.* Perhaps specify that you mean the discursive contrast here.

Page 7: Methods and field

Do you mean that you draw on data from two studies that also resulted in two other publications? Or do you only draw on the result/findings from these two published studies? Please clarify here.

Also, did you include the conversations with NGO officials in your analysis? Or did these merely interpret your analysis? This could also need clarification.

Also, could you include some of the questions (or perhaps topic guide) used for the interviews? Were they all the same or did they differ?

Page 8:

What does it add to the study to divide the groups into ‘youth’ and ‘adults’? I find it a bit confusing, also because some can go into both groups and because it is different in men. I understand that this can be an interesting point for analysis and discussion, but do not see the purposefulness in the division on this level. Also, what about those women aged 23 in the table? Even if there were no such women. Please look over the table and make it clearer.

Page 9:

Regarding education, perhaps avoid using the word ‘significantly’ as this implies a quantitative terminology and measurement.

Ethical considerations:

Do you mean that you changed some of the characteristics when providing the quotes? Or in the table? Please specify.

Page 10:

Reflexivity: in the first paragraph, I think you can reflect a little bit more about how being an insider/outsider might have influenced the responses, or at least why/how you can to the conclusion that this did not affect your data. The second paragraph of the reflexivity chapter I do not feel belong here but earlier in the methods chapter.

Please specify where you start indicating your findings.

Page 11: the wording ‘The flow of the discourse’ is used a bit lightly here and it is not clear what this means. I suggest a different, more specific, wording here. Also, how do this quote indicate that this means a change towards abandonment as opposed to change from one FGC to another? Could you provide some more evidence to support this suggestion?

Page 14: It would be great if you had a quote to support the 40-year old women who considered her extensive ‘sunna’ to be minor and harmless.

Page 15: Interesting quote by this woman. It would be good, however, to get a little bit more information about why she knows how the ‘sunna’ looks in many women – is this something the majority of women know (how it is in others) because they talk to each other or does she have special insight (being a doctor/midwife or something)?

Also, can you give a bit more detail on how ‘sunna’ is proscribed by religion? Perhaps in the background?

Page 29: What is the difference between the papers building up to the study, to this study? What can you draw on from that paper, and what comes (in result) of the present paper? Did you have different conclusions in that paper than in this one?
